# Real-World Patient-Reported Outcomes and Glycemic Results with Initiation of Control-IQ Technology

**DOI:** 10.1089/dia.2020.0388

**Published:** 2021-01-28

**Authors:** Jordan E. Pinsker, Lars Müller, Alexandra Constantin, Scott Leas, Michelle Manning, Molly McElwee Malloy, Harsimran Singh, Steph Habif

**Affiliations:** ^1^ Sansum Diabetes Research Institute, Santa Barbara, California, USA.; ^2^ University of California San Diego, Design Lab, La Jolla, California, USA.; ^3^ Tandem Diabetes Care, Data Science, San Diego, California, USA.; ^4^ Tandem Diabetes Care, Behavioral Sciences, San Diego, California, USA.

**Keywords:** Artificial pancreas, Automated insulin delivery, Glycemic control, Patient-reported outcomes, Type 1 diabetes

## Abstract

***Background:*** The t:slim X2™ insulin pump with Control-IQ™ technology, an advanced hybrid closed-loop system, became available in the United States in early 2020. Real-world outcomes with use of this system have not yet been comprehensively reported.

***Methods:*** Individuals with type 1 diabetes (T1D) (≥14 years of age) who had ≥21 days of pump usage data were invited via email to participate. Participants completed psychosocial questionnaires (Technology Acceptance Scale [TAS], well-being index [WHO-5], and Diabetes Impact and Devices Satisfaction [DIDS] scale) at timepoint 1 (T1) (at least 3 weeks after starting Control-IQ technology) and the DIDS and WHO-5 at timepoint 2 (T2) (4 weeks from T1). Patient-reported outcomes (PROs) and glycemic outcomes were reviewed at each timepoint.

***Results:*** Overall, 9,085 potentially eligible individuals received the study invite. Of these, 3,116 consented and subsequently 1,435 participants completed questionnaires at both T1 and T2 and had corresponding glycemic data available on the t:connect^®^ web application. Time in range was 78.2% (70.2%–85.1%) at T1 and 79.2% (70.3%–86.2%) at T2. PROs reflected high device-related satisfaction and reduced diabetes impact at T2. Factors contributing to high trust in the system included sensor accuracy, improved diabetes control, reduction in extreme blood glucose levels, and improved sleep quality. In addition, participants reported improved quality of life, ease of use, and efficient connectivity to the continuous glucose monitoring system as being valuable features of the system.

***Conclusions:*** Continued real-world use of the t:slim X2 pump with Control-IQ technology showed improvements in psychosocial outcomes and persistent achievement of recommended TIR glycemic outcomes in people with T1D.

## Introduction

The Tandem Diabetes Care^®^ t:slim X2™ insulin pump with Control-IQ™ technology is an advanced hybrid closed-loop system designed to help improve time in range (TIR) using continuous glucose monitoring (CGM) values to predict glucose levels 30 min ahead and adjust insulin delivery accordingly. This technology was recently approved by the U.S. Food and Drug Administration (FDA) after a 6-month randomized, controlled clinical trial, where participants showed improved TIR 70–180 mg/dL to 71%, a mean adjusted improvement of 11 percentage points compared to sensor-augmented pump in participants with type 1 diabetes (T1D).^1^ Although automated insulin delivery (AID) systems have been extensively studied in the context of T1D,^2^ prior reports mainly focused on glycemic outcomes in clinical trials, with few studies focusing on patient-reported outcomes (PROs) such as improvements in sleep and psychosocial well-being, user experience with the technology, and perceived reduction in diabetes burden.^3–5^

To date, there have been only a few reports of real-world outcomes involving large numbers of individuals with diabetes using AID systems, such as the Medtronic MiniMed 670G system.^6^ We have previously reported on real-world outcomes in people with T1D using Basal-IQ^®^ technology. Use of this particular predictive low-glucose suspend (PLGS) system led to significant reductions in hypoglycemia while also improving glycemic control with sustained use.^7,8^ Retrospective analysis of glycemic data of early adopters of Control-IQ technology has also recently been presented.^9,10^ However, there are no published reports describing the glycemic performance or PROs related to this technology in the real world.

In this study, we assessed both real-world glycemic outcomes and PROs on device-related satisfaction, diabetes impact, emotional well-being, and user acceptance after initiating Control-IQ technology.

## Methods

From March 13, 2020 through March 24, 2020, all individuals with a self-reported diagnosis of T1D, who were at least 14 years of age, and had completed either the purchase of a new Tandem t:slim X2 insulin pump with Control-IQ technology, or had performed a software update of their existing t:slim X2 insulin pump with or without Basal-IQ Technology to switch to Control-IQ technology, were invited to participate in the study by email. In addition, to allow for users to complete onboarding and experience using the new technology, only those individuals who had been using the system for at least 3 weeks at the time of the start of the study were eligible to participate. Participants completed psychosocial questionnaires at timepoint 1 (T1) (at least 3 weeks after starting Control-IQ technology) and timepoint 2 (T2) (4 weeks from T1), with the goal of determining if there were immediate and sustained effects of Control-IQ technology use. PROs and glycemic outcomes were reviewed at each timepoint.

After providing study consent online, participants completed questionnaires at T1, including demographics and various diabetes-specific items. PROs included a Control-IQ technology-specific Technology Acceptance Questionnaire (TAS)^11^ to assess positive and negative experiences with Control-IQ technology. The Control-IQ-specific version of the TAS had previously been used as part of the pivotal trial of Control-IQ technology.^1^ Scores could range from −80 to +80 with higher scores indicating greater acceptance of the specific technology. To assess emotional well-being, participants completed the well-being index (WHO-5) measure at both T1 and T2.^12^

The Diabetes Impact and Devices Satisfaction (DIDS) Scale is validated for use with individuals with T1D and evaluates users' experience interacting with their insulin delivery device, and the impact of diabetes on their life.^13^ The DIDS was also completed at both T1 and T2. Two open-ended items assessing participants' trust and satisfaction with Control-IQ technology were also included in both surveys. Automated emails were used to remind participants to complete their questionnaires at both T1 and T2.

At the time of each assessment, and later if the required data were not uploaded, participants were reminded to upload their insulin pump data to Tandem's t:connect web application for tracking insulin delivery, glucose readings, and other statistics around the use of Control-IQ technology. Participants were only included in the analysis if they had completed surveys at both T1 and T2 and had insulin pump data available in the t:connect web application at each time point.

The Western Institutional Review Board (WIRB) approved the study. Informed consent was obtained from each participant. Study participants received a $20 reward card after completing the follow-up survey. In addition to the data collected as part of this study, glycemic data (through the t:connect web application) were also available for most of the study participants from their pre-Control-IQ technology device use. Participants had previously consented to the use of their data for research purposes. These data were included in a subanalysis to evaluate the pre-and post-Control-IQ technology real-world glycemic outcomes for this subgroup of participants.

### Statistical analysis

Sensor-glucose values leading up to each survey time point were analyzed per recent international consensus statement and ADA guidelines,^14,15^ to include mean glucose, coefficient of variation, TIR 70–180 mg/dL, time >180 mg/dL, time >250 mg/dL, time <70 mg/dL, and time <54 mg/dL, with the goal of determining if individuals were achieving recommended CGM time in target ranges, and if these improvements were maintained at the second time point. Insulin delivery data included total insulin delivered, basal and bolus insulin delivered, and use of available automation activities (overall time in automation, time in sleep activity, and time in exercise activity).^16^

Two items assessing satisfaction and trust with Control-IQ technology required open-ended responses and were analyzed using content analysis. Comments were reviewed independently by three study team members with prior experience in qualitative analysis. Initial review of these comments resulted in dominant themes that were then discussed by the study team to identify primary factors affecting trust and satisfaction with participants' current insulin delivery device.

We included only participants who had uploaded at least 21 days of data before T1 and between T1 and T2 at time of the analysis. Outcomes were aggregated by mean or median depending on their distribution. TIR for example has been traditionally reported as a mean measure, but Control-IQ technology increases TIR leading to a skewed distribution that is better described by a combination of the median and the interquartile range measures. Wilcoxon-signed rank tests were performed to compare differences between baseline (T1) and follow-up (T2). Data were analyzed using the scipy.stats module in python.

## Results

### Participant characteristics

**FIG. 1.**
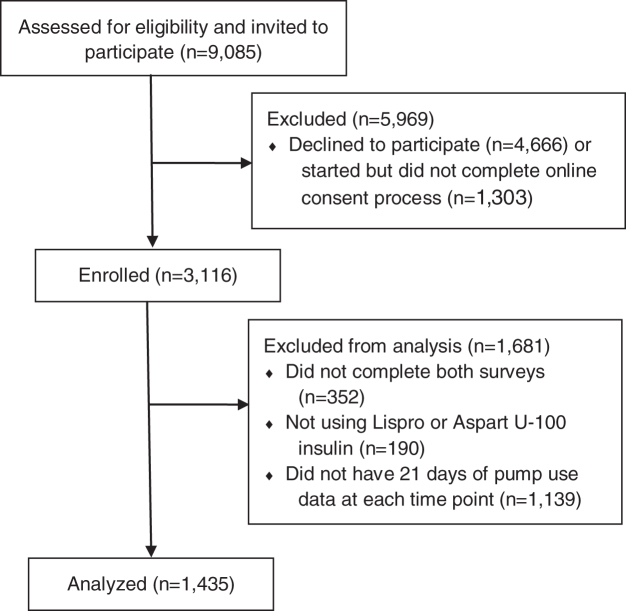
Study recruitment and analysis.

In all, 9,085 potential participants who had been using Control-IQ technology for at least 21 days received the study invitation. Of these, 3,116 individuals consented and completed enrollment and 1,435 participants (46%) completed the questionnaires at both time points (T1 and T2) (Fig. 1). The initial surveys at T1 were conducted a mean of 43.1 (±9.9) days from starting Control-IQ technology use.

Baseline demographics of study participants are listed in Table 1. Pre-Control-IQ technology glycemic data were available for most study participants (*n* = 1,127).

**Table 1.** Participant Attributes at Time point 1 (At Least 3 Weeks Using Control-IQ Technology)

Age	45.5 (16.6)
Gender	Female: 51.3% (736)
	Male: 48.6% (698)
Ethnicity	White: 89.9% (1290)
	Hispanic/Latino/Spanish: 3.7% (53)
	Other: 2.3% (33)
	Black or African American: 1.7% (24)
	Prefer not to answer: 1.4% (20)
	Asian: 0.7% (10)
	American Indian or Alaska Native: 0.3% (4)
	Unknown: 0.1% (1)
Most recent A1C	6.9% (±0.9)
Education	High school graduate: 34.7% (498)
	Bachelor's degree: 34.0% (488)
	No answer: 19.8% (284)
	Advanced degree: 7.3% (105)
	Less than high school degree: 4.2% (60)
Insurance	Other: 83.2% (1194)
	Medicare: 14.1% (202)
	Medicaid: 2.7% (39)
Diabetes duration	25.4 (15.4) years
Prior CGM use	Yes: 95.7% (1373)
	Sometimes: 2.2% (32)
	No: 2.1% (30)
Prior insulin delivery	Previous Tandem pump user: 74.3% (1066)
	Non-Tandem pump user: 19.1% (274)
	MDI: 6.6% (94)

Data presented as mean (SD) or % (*n*).

CGM, continuous glucose monitoring; MDI, multiple daily injections.

### Glycemic metrics

At timepoint 1, there was a mean of 40 therapy days of use. At timepoint 2, the mean was an additional 24 days of therapy use. Percent of time in closed-loop automation was 96%, with sleep activity in use 33% of the time (Table 2). Overall, most participants were meeting consensus statement TIR guidelines by exceeding 70% TIR 70–180 mg/dL and maintaining only 1.2% time <70 mg/dL.

**Table 2.** Glycemic Outcomes for *N* = 1,435 Study Participants (at Time point 1 vs. Time point 2). Nighttime Is Defined as 10 PM to 6 AM

	Time point 1	Time point 2	*P*
Time in range (mean)	76.7 (±11.6)	77.2 (±12.3)	<0.001
Time in range (median)	78.2 (70.2–85.1)	79.2 (70.3–86.2)	<0.001
Time in range at day (mean)	75.9 (±11.9)	76.6 (±12.4)	<0.001
Time in range at day (median)	77.6 (69.2–84.8)	78.2 (69.2–85.7)	<0.001
Time in range at night (mean)	78.4 (±13.5)	78.5 (±14.7)	0.082
Time in range at night (median)	80.7 (71.2–88.2)	82.2 (71.2–89.2)	0.082
Mean glucose	147.3 (±18.5)	146.6 (±19.9)	<0.001
Median glucose	145.0 (135.0–157.0)	144.0 (133.0–157.0)	<0.001
Coefficient of variation	31.8 (±5.2)	31.3 (±5.2)	<0.001
Time <70 mg/dL	1.3 (0.6–2.3)	1.2 (0.5–2.4)	0.818
Time <54 mg/dL	0.2 (0.1–0.4)	0.2 (0.0–0.4)	0.826
Time >180 mg/dL	19.8 (12.8–28.4)	19.0 (11.8–28.2)	<0.001
Time >250 mg/dL	2.9 (1.2–6.2)	2.5 (0.9–5.9)	<0.001
Total dose delivered	45.9 (33.7–64.4)	45.0 (33.7–63.5)	0.083
Basal insulin units	22.5 (15.9–32.3)	22.6 (16.1–32.8)	<0.001
Bolus insulin units	22.3 (15.8–32.6)	21.9 (15.5–32.3)	<0.001
Time in automation	95.7 (92.7–97.3)	96.0 (92.6–97.4)	0.722
Time in sleep activity	32.8 (27.8–37.1)	33.4 (27.2–37.6)	<0.001
Time in exercise activity	0.3 (0.0–2.2)	0.0 (0.0–2.5)	<0.05

At T1 (3 weeks of Control-IQ technology use), TIR was 78.2% (70.2%–85.1%), and then improved significantly at the time of the second survey to 79.2% (70.3%–86.2%) (*P* < 0.001). Time in hyperglycemia (both >180 and >250 mg/dL) were reduced significantly at T2 (*P* < 0.001) with no change in hypoglycemia (Table 2).

When analyzed by age group, significant improvements in overall TIR 70–180 mg/dL between T1 and T2 were seen in individuals age 60+ years, achieving a median of 80.7% overall TIR, with 80.7% TIR during the daytime and 86.1% TIR overnight. For participants in the 41–59-year age group, despite no change in the overall median TIR at T2 (78.6% vs. 78.6%), significant reductions were noted in mean sensor glucose (146.4 ± 16.9 vs. 145.5 ± 17.6, *P* < 0.01), hypoglycemia, and hyperglycemia outcomes. Daytime TIR 70–180 mg/dL improved at T2 for 14–17-year age group, from a median of 68.9% to 71.9% alongside a significant reduction in hypoglycemia %time <70 mg/dL of 1.8% (0.5–3.1) vs. 1.3% (0.4–2.4). Overall median TIR for participants in the 18–25 years and 26–40 years age group was above 72% and 77%, respectively, at T1 and maintained at T2. These results are summarized in Table 3.

**Table 3.** Glycemic Outcomes by Age Group (at Time point 1 vs. Time point 2)

Age (years)	14–17		18–25		26–40		41–59		60+	
	Time point 1	Time point 2	Time point 1	Time point 2	Time point 1	Time point 2	Time point 1	Time point 2	Time point 1	Time point 2
*N*	72	72	134	134	358	358	524	524	347	347
Overall %time 70–180 mg/dL (mean)	71.2 (12.9)	71.6 (13.9)	72.5 (12.7)	72.7 (13.4)	75.6 (12.6)	75.5 (14.1)	77.4 (11.1)	77.7 (11.1)	79.7 (9.4)	**81.1 (9.5) *P* < 0.001**
Overall %time 70–180 mg/dL (median)	68.9 (56.9–77.8)	71.9 (60.8–806)	72.8 (63.9–79.6)	72.6 (62.6–82.4)	77.1 (67.5–84.9)	77.6 (67.1–85.6)	78.6 (70.4–84.8)	78.6 (70.5–85.5)	78.8 (72.4–85.7)	80.7 (74.7–87.2)
Daytime %time 70–180 mg/dL (mean)	68.1 (13.9)	**70.0 (14.2) *P* < 0.05**	71.7 (12.7)	72.3 (13.1)	75.1 (12.9)	75.2 (14.2)	76.8 (11.3)	77.3 (11.1)	78.5 (9.7)	**79.9 (10.1) *P* < 0.001**
Daytime %time 70–180 mg/dL (median)	68.9 (56.9–77.8)	71.9 (60.8–80.6)	72.8 (63.9–79.6)	72.6 (62.6–82.4)	77.1 (67.5–84.9)	77.6 (67.1–85.6)	78.6 (70.4–84.8)	78.6 (70.5–85.5)	78.8 (72.4–85.7)	80.7 (74.7–87.2)
Nighttime %time 70–180 mg/dL (mean)	77.2 (12.9)	74.7 (15.7)	74.1 (15.1)	73.5 (16.8)	76.4 (14.4)	76.1 (16.1)	78.5 (13.4)	78.7 (14.0)	82.1 (11.4)	**83.5 (11.5) *P* < 0.001**
Nighttime %time 70–180 mg/dL (median)	80.5 (69.4–85.8)	78.4 (65.9–87.1)	77.5 (65.2–86.2)	76.7 (67.7–85.3)	79.0 (69.0–86.6)	79.6 (68.3–87.4)	81.3 (72.3–88.2)	82.4 (71.0–88.9)	84.3 (75.0–90.8)	86.1 (77.4–91.7)
Mean CGM glucose, mg/dL	154.6 (22.8)	155.5 (24.8)	152.9 (21.1)	152.6 (22.7)	148.0 (21.8)	148.2 (23.9)	146.4 (16.9)	**145.5 (17.6) *P* < 0.01**	144.4 (13.9)	**142.3 (4.2) *P* < 0.001**
Coefficient of variation	35.6 (±5.9)	33.9 (±5.4)	34.0 (±5.1)	33.4 (±5.0)	32.4 (±5.0)	31.8 (±5.2)	31.5 (±4.8)	31.2 (±4.9)	30.3 (±5.0)	29.5 (±4.9)
%Time <70 mg/dL (median)	1.8 (0.5–3.1)	**1.3 (0.4–2.4) *P* < 0.001**	1.2 (0.6–2.3)	1.2 (0.5–2.2)	1.4 (0.6–2.6)	1.3 (0.6–2.6)	1.4 (0.6–2.3)	**1.4 (0.6–2.5) *P* < 0.05**	1.0 (0.5–1.9)	1.0 (0.4–2.0)
%Time <54 mg/dL (median)	0.2 (0.1–0.6)	**0.2 (0.0–0.5) *P* < 0.05**	0.2 (0.1–0.4)	0.1 (0.1–0.4)	0.2 (0.1–0.4)	0.2 (0.1–0.4)	0.2 (0.1–0.4)	**0.2 (0.1–0.5) *P* < 0.05**	0.1 (0.0–0.3)	0.1 (0.0–0.3)
%Time >180 mg/dL (median)	25.0 (15.9–36.9)	25.1 (17.8–35.7)	23.8 (17.3–32.2)	25.0 (15.5–33.8)	21.2 (12.6–29.5)	19.9 (11.5–30.8)	18.8 (12.8–27.3)	**18.9 (12.1–27.2) *P* < 0.05**	17.2 (12.0–24.7)	**16.2 (10.6–22.9) *P* < 0.001**
%Time >250 mg/dL (median)	5.7 (2.6–13.8)	5.9 (2.2–11.5)	5.2 (2.2–8.6)	4.8 (2.2–8.4)	3.2 (1.2–6.7)	2.8 (0.8–7.3)	2.7 (1.1–5.6)	**2.5 (0.9–5.4) *P* < 0.01**	2.3 (1.0–4.5)	**1.8 (0.7–3.9) *P* < 0.001**

Nighttime is defined as 10 PM to 6 AM. Significant changes are bolded and listed with *P*-values below each entry.

For the subgroup of study participants who had at least 30 days of CGM data before and after starting Control-IQ technology (*n* = 1127), median TIR improved from 69.8% (56.7–79.8) to 79.4% (70.9–86.3) (Supplementary Table S1). Although there were also significant improvements in time <70 mg/dL and time >250 mg/dL, the main driver of the improved overall TIR was the improvement in time >180 mg/dL from 28.3% (17.5–41.9) to 19.0% (11.5–27.5) (*P* < 0.001).

### PRO analysis

The overall score for the TAS (used at T1 only) was 49.7 (±15.3) (score range = −80 to +80), demonstrating participants' endorsement and acceptance of Control-IQ technology (Table 4). The DIDS scale showed a reduction in the overall impact of diabetes on participants' lives with continued use of Control-IQ technology (2.8 ± 1.4 at T1 vs. 2.7 ± 1.4 at T2, *P* < 0.01), while also demonstrating an improvement in device-related satisfaction (9.0 ± 1.1 at T1 vs. 9.1 ± 1.1 at T2, *P* < 0.001). A significant reduction in emotional well-being scores was observed at T2 (69.8 ± 18.0 at T1 vs. 68.2 ± 17.8 at T2, *P* < 0.001).

**Table 4.** Results from Patient-Reported Outcomes at Time point 1 and Time point 2 (*N* = 1,435)

	Time point 1	Time point 2	Significance
WHO-5	69.8 (18.0)	68.2 (17.8)	*P* < 0.001
DIDS scale: diabetes impact	2.8 (1.4)	2.7 (1.4)	*P* < 0.01
DIDS scale: device satisfaction	9.0 (1.1)	9.1 (1.1)	*P* < 0.001
Technology acceptance	49.7 (15.3)	—	—

DIDS, Diabetes Impact and Devices Satisfaction.

Content analysis conducted on responses from open-ended items assessing trust and satisfaction with Control-IQ technology highlighted consistent themes across T1 and T2. Factors contributing to participants' high trust in the system included sensor accuracy, improved diabetes control, reduction in extreme blood glucose levels, and improved sleep quality. Primary themes contributing to high user satisfaction with Control-IQ technology included some overlap with the themes describing participants' trust with this technology (e.g., reduction in extreme blood glucose levels and improved diabetes control related to expected improvements in HbA1c) (Table 5). In addition, participants reported ease of use, effective connectivity to the CGM, and improved quality of life as being valuable features of the system.

**Table 5.** Open-Ended Responses: Primary Themes and Supporting Quotes from Participants

Item: How satisfied are you with your t:slim X2™ pump with Control-IQ technology?	
Primary themes	Participant quotes
1. Ease of use	*“The system was easy to learn and is easy to use on a daily basis without much change in my daily process*.” (Female, 36 years old)
2. Improved quality of life	*“This new system is a true-life changer. I have been type 1 since 2015 after having my entire pancreases removed. This is the first time since then that I feel normal…it is a life changing improvement.*” (Male, 55 years old)
3. Improved diabetes control	*“I have stayed in range much longer due to the constant automatic adjustments and mini boluses. I am also able to sleep better due to the option I have to set a ‘sleep’ activity. My mind is more at ease when control IQ is running*.” (Female, 22 years old)
4. Reduction in extreme blood glucose levels	*“The functionality of this pump is top of the market. It's assistance in eliminating highs is truly amazing and will absolutely help my A1C come down…”* (Female, 18 years old)
5. Pump integration with CGM	“*Night and day from the previous devices I have utilized. The CGM/Pump combination is a dramatic improvement over any device used in the past*.” (Male, 61 years old)
*Item: How much do you trust your t:slim X2 pump with Control-IQ technology?*	
*Primary themes*	*Participant quotes*
1. Accuracy of the sensor	“*The sensor accuracy combined with Control-IQ provides great peace of mind*.” (Male, 71 years old)
2. Improved sleep quality	“*I can sleep without worrying about highs or lows. After I installed the software every morning my bg is around 100 mg/dL. That's enough proof for me*.” (Male, 44 years old)
3. Improved diabetes control	“*It controls my BS and provides me with constant info on my progress all day long. I have been able to continue a weekly time in range percentage of 84% to 87%*.” (Male, 63 years old)
4. Reduction in extreme blood glucose levels	“….(*it) is, by far, the most intuitive pump on the market…For the first time in 24 years, it has taken away the worry I have about hypoglycemia while I sleep. There have been times where my sugar rises without my knowledge and I have watched how the algorithm works to combat the rise… I do feel that this system has helped my anxiety and allowed me to be less obsessive…I love Control IQ and I am sure my body does too.*” (Female, 33 years old)

## Conclusions

Tandem's t:slim X2 insulin pump with Control-IQ technology offers a significant advancement in diabetes care. A 6-month randomized, controlled clinical trial showed sensor glucose TIR 70–180 mg/dL improved to over 70%, with very high user satisfaction of the system.^1^ Further studies of Control-IQ technology have shown improvements in TIR due to significant reductions in hyperglycemia.^17^

In addition to glycemic improvements, ease of use is an important feature of Control-IQ technology. The t:slim X2 insulin pump pairs with a CGM (Dexcom G6; Dexcom, Inc.), and once the Control-IQ feature is activated, the user does not need to switch modes or reactivate closed-loop, as the system will automatically adjust insulin delivery as soon as valid CGM measurements are received. Fingerstick capillary blood glucose calibration measurements are not required. In the pivotal trial leading to FDA clearance of Control-IQ technology, participants performed a median of only 0.21 fingersticks per day when using the system, as the Dexcom G6 CGM used with Control-IQ technology is cleared for nonadjuvant use.^18^ Control-IQ technology is also the first FDA-cleared AID system to provide automated insulin correction boluses based on predicted CGM data.

Given these features and previously reported improvements in glycemic results from the pivotal trial, we sought to determine if real-word glycemic results and PROs matched the experience in clinical trials. Our study collected real-world glycemic data and psychosocial questionnaires on over 1,400 individuals with T1D who had been using Control-IQ technology for at least 3 weeks, and then assessed them again 4 weeks later. The glycemic improvements in this real-world data set show that individuals achieved median TIR 70–180 mg/dL >78% after 3 weeks of use of Control-IQ technology, with the age 60+ cohort achieving TIR over 80%. The highest TIR was consistently overnight.

Our previous reports have noted improvements in real-world glycemic outcomes with the addition of Basal-IQ technology (PLGS), where sensor glucose time <70 mg/dL decreased to 1.18%.^7,8^ We believe that the positive user experience of the t:slim X2 insulin pump with Basal-IQ technology facilitated these individuals with diabetes to achieve these results,^3^ results that were actually better than those seen in the pivotal PROLOG trial.^19^ However, for nonglycemic results, until now, we have only been able to offer limited reports on the software update experience, as PROs were not the focus of prior studies. In this study, we sought to better understand the overall patient experience using Control-IQ system technology and see if this experience correlated to glycemic outcomes.

In this study, we saw a high technology acceptance after 3 weeks of Control-IQ technology use (T1). Based on participants' responses top three, highest scoring (scores closer to 5 indicated better acceptance of technology) items were as follows: *I would very much like to keep using Control-IQ (technology)* (4.84 ± 0.56), *I have greater peace of mind while wearing the device* (4.68 ± 0.76), and *I feel less burdened in managing diabetes than my prior method* (of insulin delivery) (4.23 ± 1.09). Although participants reported high device satisfaction and relatively low diabetes burden at T1, a minor but statistically significant reduction in diabetes-related impact and an improvement in device-related satisfaction were noted at T2.

A significant number of these positive changes may have been related to improved glycemic control, as participants consistently reported improved glycemic control, reduction in extreme blood glucose levels, and improved sleep quality as consistent themes in their open-ended responses. Despite content analysis highlighting the theme of improved quality of life, at T2 there was a reduction in emotional well-being scores. Although this study did not ask questions specific to the COVID-19 pandemic, several participants reported quarantine-related stress as lockdowns occurred during data collection, which seemed to be a major contributor of this score reduction.

For example, a 67-year old female participant shared, “*this technology works extremely well to maintain my glucose levels in a more normal range than I have ever experienced. It is even more helpful/appreciated now that I brought my 93-year-old mother back home from the Memory Care facility she was at knowing she was at greater risk there than at home with me for getting COVID-19. Taking care of her, I have less time to care for myself, but the Control-IQ technology is managing a lot of that for me*.” Another participant reflected, “*under stay-at-home order with kids out of school and work disrupted due to COVID. Not the ideal time to survey about well-being. If I feel less than great it probably is because of all of this, and I think I would be doing better (sleep, doing interesting things) if life was a little more normal!*” (39-year-old, male). Interestingly, glycemic outcomes continued to improve despite the decreased well-being score over time, suggesting that the system functioned very well regardless of how these concerns for well-being affected individuals.

Currently, <20% of individuals with T1D achieve their ADA recommended A1c goal.^20^ Prior studies of different systems have pointed to the need to keep closed-loop automation active, with improved TIR and decreased A1c correlating with time spent with closed-loop automation active.^21^ A considerable amount of literature has been developed on how to help patients with diabetes use AID systems, particularly related to keeping closed-loop automation active.^22,23^ Control-IQ technology has only one reason for automation to cease (loss of CGM connectivity for 20 or more minutes), and automatically resumes automation as soon as valid CGM values are received with no need for the user to switch on or activate automation in any way. PROs suggesting high trust in the system related to CGM accuracy and efficient connectivity to the CGM as valuable features matches our real-world results showing 96% use of closed-loop automation. This suggests that many of these prior reported limitations of other systems are not present with Control-IQ technology.

When considering long-term use of AID, the CARES paradigm (Calculate, Adjust, Revert, Educate, and Sensor characteristics) offers a tool for diabetes clinicians and diabetes educators to help patients better understand how to use and adjust many aspects of their AID system.^24^ This is particularly important with regard to recognizing the very real limitations that still are present in every insulin pump system—the potential for infusion site failures, needing a back-up plan to deliver insulin, and what to do when a sensor is not working or not available.

Specifically related to Control-IQ technology, there have been very promising results related to initializing the system using parameters based on total daily insulin dose (“MyTDI”),^25^ although exactly how to integrate this into clinical practice is not yet clear. In addition, there is still ongoing discussion about how effective clinician-led optimization of open-loop parameters are for closed-loop use.^26^ Adolescents and young adults, who have shown improvements in their glycemic control with Control-IQ technology use,^27^ may have additional educational needs around device use that become apparent over time. Further studies will be needed to determine optimal patient teaching methods and the clinician's role in managing this specific AID system.

We recognize limitations in our analysis. This was an observational study with no control group. Early adopters of the technology may be more likely to report positive impressions. In addition, there is the possibility of significant selection bias, as only 3,116 of the potential 9,138 eligible individuals with at least 21 days of Control-IQ technology use consented to participate in the study, and only 1,435 subjects had adequate data for analysis at both survey time points. Given 54% of the 3,116 individuals who completed informed consent did not complete the second survey, the completed results and glycemic outcomes may be biased toward individuals who were more engaged with their use of the system. In addition, the study was performed as lockdowns for COVID-19 were occurring throughout the United States, potentially affecting study participation and survey response rates. Despite these limitations, and even considering participants had a relatively higher TIR (69.8%) before adopting Control-IQ technology, the improvement in TIR from prior therapy in our study (∼10%) was very similar to that seen in prior trials,^1^ suggesting these results may be generalizable to the larger population of individuals with T1D.

In conclusion, continued real-world use of Tandem's Control-IQ technology showed improvements in psychosocial outcomes and persistent achievement of recommended TIR glycemic outcomes with continued use for at least 7 weeks in over 1,400 individuals. Given the very positive perception of the system from PROs, we expect that individuals with T1D will continue to use the system over time, as they consistently emphasized ease of use, effective connectivity to the CGM, improved glycemic control, and improved quality of life as important factors related to trust and satisfaction with the system.

## Supplementary Material

Supplemental data
