# Supplementary material for: Real-World Patient-Reported Outcomes and Glycemic Results with Initiation of Control-IQ Technology
Source: Diabetes Technol Ther. 2021 Jan 28;23(2):120–7. doi: 10.1089/dia.2020.0388 (PMC7868573; doi:10.1089/dia.2020.0388)
Supplement: Supplemental data [file Supp_Table1.pdf]

Supplemental Table 1. Glycemic outcomes pre- and post- Control-IQ technology use for the subgroup of study participants who had at least 30 days of CGM data prior to and after starting Control-IQ technology (n=1,127).

	**Pre-Control-IQ**	**Post-Control-IQ**	**p value**
Time in Range 70-180 mg/dL (Mean)	67.0 (±17.0)	77.5 (±12.2)	<0.001
Time in Range 70-180 mg/dL (Median)	69.8 (56.7-79.8)	79.4 (70.9-86.3)	<0.001
%Time <70mg/dL	1.3 (0.5-2.7)	1.2 (0.5-2.4)	<0.001
%Time <54mg/dL	0.2 (0.0-0.4)	0.2 (0.0-0.4)	NS
%Time >180 mg/dL	28.3 (17.5-41.9)	19.0 (11.5-27.5)	<0.001
%Time >250 mg/dL	5.5 (2.1-12.1)	2.5 (0.8-5.7)	<0.001
